# Severe graft‐versus‐host disease post allogeneic hematopoietic stem cell transplantation due to loss of HLA heterozygosity in recipient lymphocytes after full graft rejection

**DOI:** 10.1002/cai2.72

**Published:** 2023-04-18

**Authors:** Song Xue, Lili Miao, Zimu Gong, Wenqiu Huang, Yongping Zhang, Fuhong Liu, Jingbo Wang

**Affiliations:** ^1^ Department of Hematology, Aerospace Center Hospital Peking University Aerospace School of Clinical Medicine Beijing China; ^2^ Beijing Bo Fu Rui Gene Diagnostics Co., Ltd Beijing China; ^3^ Cancer Center, Houston Methodist Hospital Houston Texas USA

**Keywords:** chimerism, germ cell tumors, graft‐versus‐host disease, heterozygosity, stem cell transplantation

## Abstract

Germ cell tumors complicated by hematological malignancy (HM) are a rare clinical phenomenon. Allogeneic hematopoietic stem cell transplantation (allo‐HSCT) is a potentially effective therapy, but graft‐versus‐host disease (GVHD) is a life‐threatening complication. We report a case of a 13‐year‐old female patient diagnosed with germ cell tumors followed by acute lymphoblastic leukemia. After chemotherapy, she received allo‐HSCT and her chimerism rate decreased rapidly to near zero by 6 months without evidence of HM recurrence. However, she developed severe, multiorgan GVHD‐like manifestations. DNA analysis revealed the pathogenesis of GVHD to be loss of HLA heterozygosity in recipient hematopoietic cells.

## Graphical abstract

This paper presents a unique case of a 13‐year‐old female patient diagnosed with concomitant germ cell tumors and acute lymphoblastic leukemia, who underwent allogeneic hematopoietic stem cell transplantation and subsequently developed severe graft‐versus‐host disease (GVHD)‐like manifestations (hereon, we call GVH‐like‐disease, GVHLD) despite the absence of donor chimerism and recurrent hematological malignancy. The underlying etiology was found to be HLA loss of heterozygosity (HLA‐LOH) in the patient's newly reconstituted hematopoietic lineage. This is the first report of fatal GVHLD caused by HLA‐LOH in the recipient's own immune system without donor chimerism. The paper highlights the importance of understanding the role of HLA‐LOH in GVHLD pathogenesis.

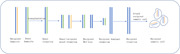

**Abbreviations**
ALL: acute lymphoblastic leukemia
GCT: germ cell tumor
GVHD: graft‐versus‐host disease
HSCT: hematopoietic stem cell transplantation

## 1 INTRODUCTION

Germ cell tumors (GCTs) are malignancies originating from primordial and pluripotent germ cells. Nonseminoma GCTs of the mediastinum have a unique clinical presentation and pose a high risk for the development of concomitant hematological malignancy (HM) [1, 2, 3]. In patients with concomitant GCTs and HM, the median time between GCT diagnosis and HM development is 6 months, and in 30% of patients, both malignancies occur simultaneously [4, 5]. The most common subtype of HM is acute megakaryoblastic leukemia, whereas other types of leukemias including acute lymphoblastic leukemia (ALL) have also been reported [4, 6, 7]. A possible explanation for the co‐occurrence of these malignancies is that GCTs and concomitant HM may arise from the same precursor cell, considering that pluripotent primordial germ cells and hematopoietic stem cells both originate from the yolk sac [8, 9]. Conventional therapy for GCTs complicated by HM has limited efficacy with patients having a median survival of only 5 months after diagnosis of HM. Many studies have reported that allogeneic hematopoietic stem cell transplantation (allo‐HSCT) prolongs survival of some GCT/HM patients [4, 10, 11].

Graft‐versus‐host disease (GVHD) is a common complication of allo‐HSCT mediated by donor‐derived lymphocytes attacking recipient tissues because of mismatched HLA expression. It is commonly accepted that the pathogenesis of GVHD depends entirely on successful donor chimerism. Here, we present a special case of a patient diagnosed first with GCTs and then ALL shortly afterward, who received allo‐HSCT and then developed persistent severe GVHD‐like manifestations despite the absence of donor chimerism and recurrent HM cells. The etiology of such contradicting clinical phenomena was eventually found to involve HLA loss of heterozygosity in the newly reconstituted hematopoietic lineage.

## 2 CASE PRESENTATION

A 13‐year‐old Chinese female, with no significant past medical or surgical history, presented with abdominal distension and pain in January 2019. Ultrasound findings included a naive uterus and mixed masses in bilateral adnexa. The patient had an ɑ‐fetoprotein (AFP) level of 4833 ng/mL and a human chorionic gonadotropin level of 56.51 IU/ml. Exploratory laparotomy was performed on January 26, 2019. Pathology showed that the tumor in the ovary was a mixed malignant GCT. The patient underwent one cycle of chemotherapy with the BEP regimen (bleomycin, 15 mg/d D1–3; etoposide, 100 mg/d D1–2; cisplatin 30 mg/d D1–5) in February, 2019.

Serum AFP decreased to 10 ng/mL after surgery and the first cycle of chemotherapy. Thrombocytopenia and significant leukocytosis with a leukocyte count as high as 26.49 × 10^9^/L had developed after chemotherapy. Bone marrow aspiration was performed on March 21, 2019, and confirmed the diagnosis of B‐cell ALL, chromosome karyotype: 47, XY, +14[20]. The patient was treated with VDLP (vincristine, daunorubicin, l‐asparaginase, and prednisone), high‐dose methotrexate (MTX), and CAM (cyclophosphamide, cytarabine, and mercaptopurine) chemotherapy from April to July 2019. On August 8, 2019, repeat bone marrow aspiration showed complete remission. Twelve days later on August 20, 2019, ultrasound scans showed severe splenomegaly (19.8 cm × 4.4 cm). On August 22, 2019, repeat bone aspiration showed relapse with flow cytometric analysis, indicating that abnormal B lymphoblasts accounted for 14% of cells. Oral trametinib was started in September 2019 and continued until January 2020. On December 5, 2019, a pelvic and abdominal computed tomography (CT) scan revealed an irregular mass of soft tissue in the pelvis and abdomen, measuring 13.2 cm × 8.3 cm × 20.5 cm. The AFP level at this time was 18 293 ng/mL, suggesting GCT recurrence. She underwent resection of the pelvic mass along with splenectomy and partial omentectomy on December 17, 2019. Pathology showed that the right adnexa tissue and mass were an immature teratoma Grade 3) and immunohistochemical staining revealed B lymphocytic leukemia/lymphoma infiltration of the spleen. The patient then received four additional cycles of chemotherapy with the BEP regimen from December 2019 to March 2020.

On April 13, 2020, repeat bone marrow aspiration showed 30% lymphoblasts. Flow cytometric analysis indicated that 9.17% of the cells were CD19+, CD45dim+, CD22+, cCD79a+, CD34+, CD10dim+, CD38+, and CD20 partially+, which was consistent with abnormal B lymphoblasts. The karyotype was 47, XY, +14 [19]. She then received three cycles of VICP (vincristine, idarubicin, cyclophosphamide, and prednisone) chemotherapy and one cycle of HR‐3 (dexamethasone, cytarabine, etoposide, and l‐asparaginase) chemotherapy from April to July 2020. On August 25, 2020, bone marrow aspiration showed morphological remission and flow cytometric analysis produced negative findings for minimal residual disease. The karyotype remained 47, XY, +14 [20].

The patient received an myeloablative conditioning regimen including total body irradiation/idarubicin/cytarabine/cyclophosphamide/antithymocyte globulin‐Fresenius S starting on August 29, 2020. The detailed process was as follows: total body irradiation at 200 cGy bid/d on Days −10, −9, and −8; idarubicin, 8 mg/m^2^/d on Days −10, −9, and −8; cytarabine, 4 g/m^2^/d on Days −7 and −6; cyclophosphamide, 1.8 g/m^2^/d on Days −5 and −4; antihuman thymocyte immunoglobulin‐Fresenius S (Astellas Pharma Inc.), 5 mg/kg/d on Days −5, −4, −3, and −2. On September 8, 2020, the patient received allo‐HSCT. Peripheral blood stem cells were provided by the father as an HLA‐haploidentical donor (blood type B for A) and she was infused with 8.8 × 10^8^/kg mononuclear cells and 3.95 × 10^6^/kg CD34+ cells. She received cyclosporin A, mycophenolatemofetil, and short‐term MTX as prophylaxis for GVHD. Neutrophil and platelet engraftment occurred on Days +18 and +24, respectively. On day +21, bone marrow morphology showed remission with no minimal residual disease detected by flow cytometry. The donor chimeric rate was 99.3%.

The patient developed cytomegalovirus viremia on Day +40 and Epstein–Barr viremia on Day +43, and after treatment with rituximab and other antiviral therapies, the virus became negative. Bone marrow aspiration was repeated on day +63, and bone marrow morphology continued to show remission with no minimal residual disease detected by flow cytometry. However, the donor chimeric rate was reduced to 64%. On Day +66, the patient developed fever and diarrhea with gastrointestinal bleeding, which was confirmed by colonoscopy and mucosal biopsy as GVHD and improved after treatment with 2 mg/kg methylprednisolone and basiliximab. Bone aspiration repeated on Day +76 showed 99.5% donor chimerism.

On Day +141, the patient presented with chest tightness, dyspnea, and hypoxemia. Chest CT showed multiple ground glass opacities in both lungs. Bronchoscopy and bronchioalveolar lavage showed no overt infection. Immune‐mediated lung injury was suspected, and the symptoms and imaging findings improved significantly after methylprednisolone treatment. Follow‐up bone marrow aspiration on Day +155 continued to show remission with negative minimal residual disease by flow cytometry, but the donor chimerism rate was only 9%. Considering the previous transplantation complications, active bone marrow hematopoiesis with good compensation, and a normal leukocyte count, no specialized treatment was administered. The patient had significant jaundice attributed to blood‐type conversion. On Day +183, bone marrow aspiration showed remission with no minimal residual disease by flow cytometry, but the donor chimerism rate was further reduced to 4% and the donor chimerism rate was 0% in peripheral blood CD3+ T cells. On Day +185, the patient developed fever, hypoxemia, hemoptysis, and diarrhea with gastrointestinal bleeding, and chest CT showed bilateral ground glass opacities (Supporting Information: Figure 1). Bronchial alveolar lavage again showed no overt infectious etiology. Diarrhea and gastrointestinal bleeding confirmed by colonoscopy and mucosal biopsy was GVHD, and she was treated with methylprednisolone again. The patient developed complete graft rejection but experienced persistent severe GVHD‐like manifestations as evidenced by lung injury, hyperbilirubinemia (blood‐type conversion had been completed), severe cardiac insufficiency, and gastrointestinal bleeding. The patient died of severe multiple organ failure on Day +198. To investigate her unique clinical course, we analyzed DNA samples.

**Figure 1:**
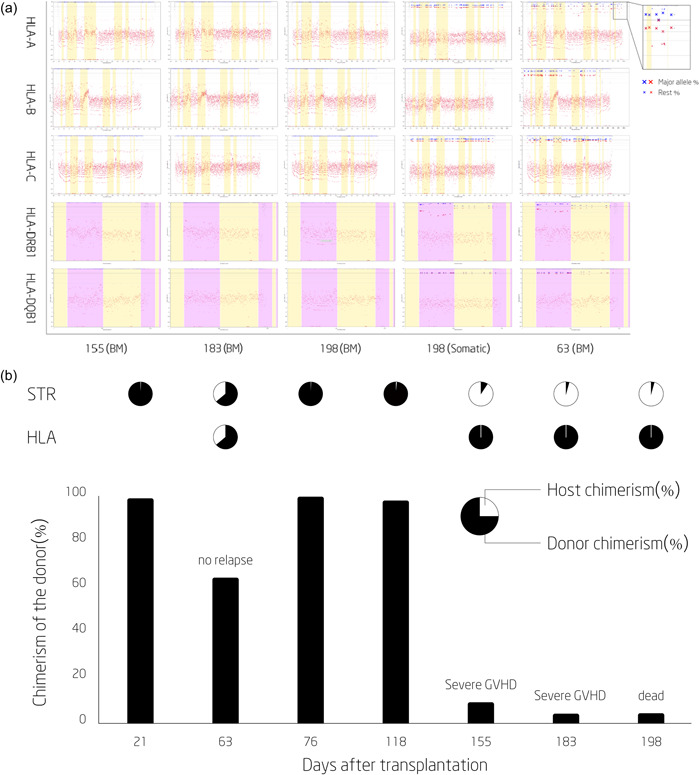
HLA loss detected by HLA typing using next‐generation sequencing (NGS) and chimerism detected by short tandem repeat (STR) and HLA typing using NGS. (a) HLA typing performed by NGS typing. BM, bone marrow; PB, peripheral blood. For HLA Class I, white bands represent introns and yellow bands represent exons. For HLA Class II loci, both yellow and pink bands represent exons, and introns are not shown. Large X's (blue and red) indicate a major allele with a higher percentage and small x's (blue and red) indicated other alleles with a lower percentage. A lack of X or x indicates homozygous. From this figure, the patient experienced HLA loss from Day +156 posttransplantation and no HLA loss was detected in the somatic sample and Day +64 sample. (b) At 64 days after allogeneic stem cell transplantation (allo‐SCT), 64% donor chimerism was detected without relapse. Pie charts above the graphs show the percentages of donor and host chimerism, as assayed by genomic HLA typing (bottom row) or STR amplification (top row). The patients eventually had >90% chimerism from Day +155 after transplantation, but the patient's HLA was not detected with HLA typing (red boxes) due to HLA loss.

HLA analysis of a Day +63 bone marrow specimen showed a normal heterozygous HLA genotype with a decreased chimerism rate (Table 1). HLA loss of heterozygosity (HLA‐LOH) was identified in Days +155 and +183 bone marrow specimens. Interestingly, a Day +198 buccal swab specimen showed normal heterozygous HLA but the peripheral blood specimen from the same day again showed HLA‐LOH (Figure 1 and Supporting Information: Table 1). Whole exome sequencing analysis revealed the NRAS exon 2: c.35G>A:p.G12D mutation and TP53 exon 4:c.215C>G:p.P72R mutation. Written informed consent for publication of this report and accompanying images was provided by the patient's parents.

**Table 1:** Chimerism detected by STR analysis.

Date of sample collection	Days after transplantation	Host chimerism (%)	Donor chimerism (%)
2020/9/29	21	0.7	99.3
2020/11/10	63	36.0	64.0
2020/11/23	76	0.5	99.5
2021/1/4	118	1.6	98.4
2021/2/10	155	91.0	9.0
2021/3/10	183	96.0	4.0
2021/3/25	198	96.0	4.0

Abbreviation: STR, short tandem repeat.

## 3 DISCUSSION

Concomitant nonseminomatous GCT with leukemia is a very rare phenomenon and it is challenging for clinicians to differentiate secondary leukemia from concomitant leukemia. The time interval between the onset of the two diseases, the cytogenetic findings, and prior chemotherapy are often helpful to differentiate these two clinical entities. In the present case, considering the short time interval between GCT and ALL diagnoses, the low cumulative etoposide dose, and the lack of characteristic karyotype findings,[12] we believed this case was best classified as concomitant leukemia with nonseminomatous GCT.

GVHD pathogenesis is highly dependent on donor chimerism. In our patient, the chimerism rate decreased to an undetectable level on Day +155 posttransplantation, but severe GVHD‐like manifestations persisted. HLA‐LOH of all Class I and II genes was identified in recipient hematopoietic cells, but not in her buccal mucosa sample collected at the same time. This is believed to be the consequence of acquired uniparental disomy of a large genomic region on chromosome 6p,[13] encompassing all HLA Class I and II loci. Therefore, we speculate that her GVHD‐like syndrome resulted from such HLA‐LOH in recipient lymphocytes, leading to recognition of her own normal tissue as foreign antigens. To our knowledge, this is the first report of fatal GVHD caused by loss of HLA heterozygosity in the recipient's own immune system without donor chimerism.

HLA‐LOH is a known mechanism for immune escape leading to leukemia relapse after SCT, in which leukemic cells escape the donor‐derived immune system by losing expression of mismatched HLA molecules [14]. HLA loss is essentially a genomic instability event, and its occurrence is not restricted to leukemia patients with posttransplantation relapse [15]. This phenomenon is rarely observed in nonmalignant cell lineages. In the present case, the early development of two different types of malignancies, clonal karyotypes, and the presence of TP53 and NRAS [16] mutations, and 14 chromosomal abnormalities indicated excessive genomic instabilities. Under the selection pressure of the donor‐derived immune system, hematopoietic cells with HLA‐LOH may have obtained a survival advantage over the residual leukemic cells, which retained the heterozygous HLA genotype. As a result, her HLA homozygous immune cells were able to reject engrafted donor cells successfully and at the same time began immunological attack of her own normal tissues, resulting in severe GVHD manifestations. The inability of donor cells to eradicate recipient lymphocytes with HLA‐LOH is certain to lead to persistent severe GVHD symptoms and death.

## AUTHOR CONTRIBUTIONS

**Song Xue**: Conceptualization (lead); data curation (lead); formal analysis (lead); investigation (lead); methodology (lead); project administration (lead); writing—original draft (lead). **Lili Miao**: Data curation (equal); methodology (equal); writing—original draft (supporting). **Zimu Gong**: Data curation (supporting); methodology (supporting); writing—review & editing (lead). **Wenqiu Huang**: Investigation (supporting); writing—original draft (supporting). **Yongping Zhang**: Investigation (supporting); writing—original draft (supporting). **Fuhong Liu**: Investigation (supporting); writing—original draft (supporting). **Jingbo Wang**: Project administration (equal); supervision (equal).

## CONFLICT OF INTEREST STATEMENT

The authors declare no conflict of interest.

## ETHICS STATEMENT

This retrospective study was approved by the Ethics Committee of Aerospace Center Hospital (No. 20211216‐XYZZ‐01).

## INFORMED CONSENT

Written informed consent for publication of this report and accompanying images was provided by the patient's parents.

## Supporting information

Supporting Information.

Supporting Information.

## Data Availability

Data openly available in a public repository that issues data sets with DOIs.
