# Supplementary figures and images for: Severe graft‐versus‐host disease post allogeneic hematopoietic stem cell transplantation due to loss of HLA heterozygosity in recipient lymphocytes after full graft rejection
Source: Cancer Innov. 2023 Apr 18;2(4):312–7. doi: 10.1002/cai2.72 (PMC10686147; doi:10.1002/cai2.72)

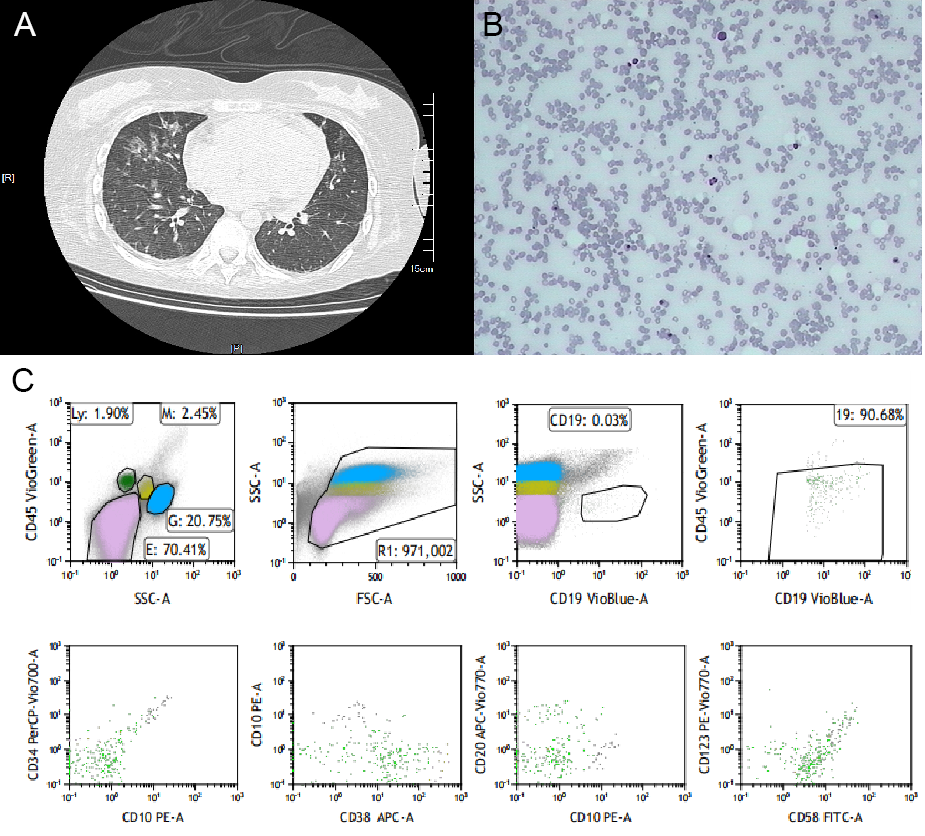

Supplement: Supplementary file 1 — Supporting Information. [file CAI2-2-312-s002.tif]
