# Supplementary material for: Severe graft‐versus‐host disease post allogeneic hematopoietic stem cell transplantation due to loss of HLA heterozygosity in recipient lymphocytes after full graft rejection
Source: Cancer Innov. 2023 Apr 18;2(4):312–7. doi: 10.1002/cai2.72 (PMC10686147; doi:10.1002/cai2.72)
Supplement: Supplementary file 2 — Supporting Information. [file CAI2-2-312-s001.doc]

**Supplementary Table 1. HLA typing for donor and patient as performed on blood samples and somatic samples**

| **Date of sample collection** | **Days after transplantation** | **Host chimerism (%)** | **Allele** | **A*** | **B*** | **C*** | **DRB1*** | **DQB1*** |
| --- | --- | --- | --- | --- | --- | --- | --- | --- |
| 2020/4/27  Donor | / | / | #3 | **02:01:01:01** | **40:01:02:01** | **07:02:01:01** | **08:03:02:01** | **06:01:01:01** |
|  |  |  | #4 | 33:03:01:01 | 58:01:01:01 | 03:02:02:01 | 04:04:01 | 03:02:01 |
| 2021/3/25  Patient | **198** | **Somatic (-)** | #1 | **02:01:01:01** | **40:01:02:01** | **07:02:01:01** | **08:03:02:01** | **06:01:01:01** |
|  |  |  | #2 | *11:01:01:01* | *40:01:02:01* | *01:02:01:01* | *04:06:01* | *03:02:01:01* |
| 2021/3/25  Patient | **198** | **PB (96%)** | #1 | **02:01:01:01** | **40:01:02:01** | **07:02:01:01** | **08:03:02:01** | **06:01:01:01** |
|  |  |  | #2 | **02:01:01:01** | **40:01:02:01** | **07:02:01:01** | **08:03:02:01** | **06:01:01:01** |
| 2021/3/10  Patient | **183** | **BM (96%)** | #1 | **02:01:01:01** | **40:01:02:01** | **07:02:01:01** | **08:03:02:01** | **06:01:01:01** |
|  |  |  | #2 | **02:01:01:01** | **40:01:02:01** | **07:02:01:01** | **08:03:02:01** | **06:01:01:01** |
| 2021/2/10  Patient | **155** | **BM (91%)** | #1 | **02:01:01:01** | **40:01:02:01** | **07:02:01:01** | **08:03:02:01** | **06:01:01:01** |
|  |  |  | #2 | **02:01:01:01** | **40:01:02:01** | **07:02:01:01** | **08:03:02:01** | **06:01:01:01** |
| 2020/11/10  patient | 63 | **BM (36%)** | #1 | **02:01:01:01** | **40:01:02:01** | **07:02:01:01** | **08:03:02:01** | **06:01:01:01** |
|  |  |  | #2 | *11:01:01:01* | *40:01:02:01* | *01:02:01:01* | *04:06:01* | *03:02:01:01* |
|  |  |  | #3 | **02:01:01:01** | **40:01:02:01** | **07:02:01:01** | **08:03:02:01** | **06:01:01:0101** |
|  |  |  | #4 | 33:03:01:01 | 58:01:01:01 | 03:02:02:01 | 04:04:01 | 03:02:01: |

#1 and #2 represent the two haploids of the patient, #3 and #4 represent the two haploids of the donor.

Lost sites and special haploids for the patient are indicated in italics. Donor and patient matched haploids in indicated in bold.

The patient had eventually >90% chimerism from day +155 post-transplantation, but the patient’s unique haploid was not detected due to HLA loss.
